# Sex and Genotype Affect Mouse Hippocampal Gene Expression in Response to Blast-Induced Traumatic Brain Injury

**DOI:** 10.1007/s12035-025-04879-5

**Published:** 2025-04-03

**Authors:** Kathleen E. Murray, Arun Reddy Ravula, Victoria A. Stiritz, Tara P. Cominski, Vedad Delic, Caralina Marín de Evsikova, Kakulavarapu V. Rama Rao, Namas Chandra, Kevin D. Beck, Bryan J. Pfister, Bruce A. Citron

**Affiliations:** 1 Laboratory of Molecular Biology, Research & Development, U.S. Department of Veterans Affairs, VA New Jersey Health Care System, East Orange, NJ USA; 2 Neurobehavioral Research Laboratory, Research & Development, U.S. Department of Veterans Affairs, VA New Jersey Health Care System, East Orange, NJ USA; 3 School of Graduate Studies, Rutgers Health, Newark, NJ USA; 4 Molecular Neurotherapeutics Laboratory, Department of Neuroscience, Mayo Clinic, Jacksonville, FL USA; 5 Department of Pharmacology, Physiology & Neuroscience, Rutgers-New Jersey Medical School, Newark, NJ 07101 USA; 6 Division of Life Sciences, School of Arts and Sciences, Rutgers University, New Brunswick, NJ USA; 7 Epigenetics and Functional Genomics Laboratory, Research & Development, U.S. Department of Veterans Affairs, Bay Pines VA Healthcare System, Bay Pines, FL USA; 8 Department of Molecular Medicine, University of South Florida, Tampa, FL USA; 9 Center for Injury Biomechanics, Materials, and Medicine, Department of Biomedical Engineering, New Jersey Institute of Technology, Newark, NJ USA

**Keywords:** Traumatic brain injury, Blast, Hippocampus, Sex differences, Gene expression, RNA-seq

## Abstract

Blast-induced traumatic brain injury (bTBI) has been identified as an increasingly prevalent cause of morbidity and mortality in both military and civilian populations over the past few decades. Functional outcomes following bTBI vary widely among individuals, and chronic neurodegenerative effects including cognitive impairments can develop without effective diagnosis and treatment. Genetic predispositions and sex differences may affect gene expression changes in response to bTBI and influence an individual’s probability of sustaining long-term damage or exhibiting resilience and tissue repair. Male and female mice from eight genetically diverse and distinct strains (129S1/SvImJ, A/J, C57BL/6J, CAST/EiJ, NOD/ShiLtJ, NZO/HlLtJ, PWK/PhJ, WSB/EiJ) which encompassed 90% of the genetic variability in commercially available laboratory mice were exposed to a single bTBI (180 kPa) using a well-established shock tube system. Subacute changes in hippocampal gene expression due to blast exposure were assessed using RNA-seq at 1-month post-injury. We identified patterns of dysregulation in gene ontology terms and canonical pathways related to mitochondrial function, ribosomal structure, synaptic plasticity, protein degradation, and intracellular signaling that varied by sex and/or strain, including significant changes in genes encoding respiratory complex I of the electron transport chain in male WSB/EiJ mice and the glutamatergic synapse across more than half of our groups. This study represents a multi-level examination of how genetic variability may influence response to bTBI and provides a foundation for the identification of potential therapeutic targets that could be modulated to improve the health of Veterans and others with histories of blast exposures.

**Supplementary Information:**

The online version contains supplementary material available at 10.1007/s12035-025-04879-5.

## Introduction

Traumatic brain injury (TBI) has emerged as the signature injury of the military conflicts in Iraq and Afghanistan [1]. Over the past two decades, blast exposure has become the most common cause of mild TBI in deployed military personnel due to the use of improvised explosive devices (IEDs) and heavy weaponry with increased explosive force [2–7]. Primary blast-induced traumatic brain injury (bTBI) occurs when exposure to the shock wave generated by a nearby explosion results in damage to the brain [8, 9]. Longitudinal studies have shown that combat veterans who sustained mild bTBIs fare poorly on measures of global disability, psychiatric symptoms, and neurobehavioral impairment when compared to combat-deployed controls, even five years after injury [10–12]. Despite these potentially significant impacts on overall quality of life, there remains no effective treatment to protect against the chronic cognitive deficits observed in TBI patients, and the molecular processes distinctly impacted by primary bTBI require further understanding. Moreover, highly variable patterns of recovery among veterans and others who have sustained similar types of TBI remain unaddressed [13–15].

Genetic differences between individuals have been proposed as a significant influence on patient outcomes following TBI, particularly in genes contributing to pre-existing risk factors, response to neurotrauma, cellular repair and plasticity, pre- and post-injury neurocognitive capacity, and epigenetic factors [16–20]. TBI leads to significant neuronal damage that contributes to the functional consequences of the injury. Adverse clinical outcomes of TBI result from a two-step process that involves both primary and secondary injuries [21]. The primary aspect of TBI is directly associated with mechanical damage resulting in a breakdown of cell membrane integrity, which leads to massive disruption of ionic homeostasis and rapid cell death [22]. Secondary injuries occur post-insult via multiple parallel, interacting, and independent signaling cascades activated in response to the primary injury that further contribute to neurodegeneration [23]. Enhanced characterization of the genetic variability that can result in variable recovery from similar injuries is essential for a deeper understanding of the complex mechanisms driving post-TBI pathology.

The identification and mechanistic understanding of specific gene polymorphisms that drive susceptibility or resistance to damage following bTBI are of the utmost importance in developing targeted treatments to improve patient outcomes. Rodent models of bTBI are apt for this task due to their considerable genetic similarity to humans and well-characterized genotypes and phenotypes. Historically, rodent models of neurotrauma have used common inbred laboratory strains, such as C57BL/6 mice and Sprague Dawley (SD) or Long Evans rats, or transgenic models to examine the effects of injury on well-known human risk factor genes (e.g., *APOE*, *BDNF*, *COMT*, *TNFR*) [24–27]. Although controlling for genotype or examining the isolated effects of targeted mutations improves reproducibility in preclinical TBI, neither approach employs a comprehensive nor representative model of the genetically diverse patient populations who are at risk for TBI [28]. Additionally, most of these studies have investigated mechanical TBIs. While this work has provided valuable insight into gene regulation following TBI and susceptibility to brain injury, more research is needed on genetic influences on bTBI specifically. Conversely, human studies present confounding variables such as patient history of other mechanisms of neurotrauma, particularly in military populations [29].

The availability of numerous mouse strains introduces an important variable that may influence functional outcomes following TBI. Strain differences have been reported in several models of brain injury, including the fluid percussion injury (FPI), controlled cortical impact (CCI), and weight drop models of TBI [28, 30, 31], stroke [32], and chronic stress [33–37]; a recent study even noted sub-strain related differences in brain damage due to hypoxic-ischemic injury in C57BL/6 mice from different vendors [38]. Sexual dimorphisms are also an essential consideration for many diseases influenced by a dysregulated immune system, including TBI [39]. For example, following CCI, male mice suffer a more aggressive neuroinflammatory response, while females have a delayed anti-inflammatory response as late as 30 days post-injury [40]. To better understand molecular processes, behavioral deficits, and recovery after bTBI, we need to consider how various mouse strains respond to injury and how these responses can be translated to humans.

The Collaborative Cross (CC) model was designed to develop outbred mice with genetic heterogeneity from eight founder strains, which encompass > 90% of genetic diversity within commercially available laboratory mice across three substrains: *M. musculus domesticus* (129S1/SvImJ, A/J, C57BL/6J, NOD/ShiLtJ, NZO/HlLtJ, WSB/EiJ), *M. musculus musculus* (PWK/PhJ), and *M. musculus castaneus* (CAST/EiJ) [41–43]. Here, we utilized the CC founder strains to examine the effects of genotype and sex on subacute changes in hippocampal gene expression at 1-month post-injury following single blast exposure (180 kPa) in male and female mice with varying genetic backgrounds. We modeled blast exposure using a well-validated shock tube system [44–48]. This study provides comprehensive data that will allow us to identify candidate genes that respond to the blast exposure that could be involved in neurodegeneration and recovery. Data obtained from this study may be used to develop further investigations into genes of interest that will lead to targeted treatments for blast TBI and generate a repository of data regarding strain variability in mouse models of blast-induced TBI.

## Materials and Methods

### Subjects

Male and female 129S1/SvlmJ (RRID:IMSR_JAX:002448), A/J (RRID:IMSR_JAX:000646), CAST/EiJ (RRID:IMSR_JAX:000928), C57BL/6J (RRID:IMSR_JAX:000664), NOD/ShiLtJ (RRID:IMSR_JAX:001976), NZO/HlLtJ (RRID:IMSR_JAX:002105), PWK/PhJ (RRID:IMSR_JAX:003715), and WSB/EiJ (RRID:ISMR_JAX:001145) mice (6 weeks of age, *n* = 3/group, 96 total mice) were obtained from The Jackson Laboratory (Bar Harbor, ME). Power analysis was conducted using G*Power (version 3.1.9.6) and indicated that a sample size of n = 3/group, power = 0.90, and alpha = 0.05, two-tailed, corresponded to an effect size = 3.59. Animals were single-housed in a temperature- and humidity-controlled environment (22 °C ± 0.5 °C, ~ 55%) with a 12-h light/dark cycle. Animals were allowed a 7-day acclimation period prior to blast exposure. Food and water were available ad libitum throughout the study for all animals. All animal experiments were performed in accordance with the guidelines of the institution and the National Institutes of Health Guide for the Care and Use of Laboratory Animals and approved by the East Orange VA Institutional Animal Care and Use Committee.

### Blast-Induced Traumatic Brain Injury

Blast injuries were performed at 8 weeks of age using a well-characterized shock tube model in the Center for Injury Biomechanics, Materials and Medicine at the New Jersey Institute of Technology as described previously [44–48]. Both sham and injured animals were anesthetized with 5% isoflurane for 5 min and fitted with silicone earplugs to prevent tympanic membrane damage [49]. bTBI mice were exposed to a single blast at 180 ± 5 kPa peak overpressure (duration: 6.5 ± 0.5 ms, impulse: 320 ± 20 kPa·msec) [47]. Sham animals were placed 5–6 ft perpendicular from the center of the shock tube and were exposed to the acoustic wave but not the shock wave generated by the blast. Pressure waves profiles within the chamber were recorded for each blast to ensure that the injury model was consistent across all animals.

### RNA Isolation

Mice were euthanized 1-month post-injury via live decapitation. Whole brains were immediately extracted, and hippocampal tissue from each hemisphere was dissected and snap-frozen on dry ice. All fresh frozen tissue samples were stored at −80 °C until use. Total cellular RNA was isolated using an RNeasy Mini Plus Kit (QIAGEN, Hilden, Germany) according to the manufacturer’s instructions. Tissue was homogenized in RLT Plus Buffer with a Polytron homogenizer (Kinematica USA, Bohemia, NY) for 45 s. RNA samples were stored at −80 °C until use and transported on dry ice for processing.

### RNA-Seq

RNA quality control and sequencing were performed by the Genomics Center at Rutgers-New Jersey Medical School. The integrity of total cellular RNA was confirmed with a Bioanalyzer 2100 (Agilent Technologies, Santa Clara, CA). Samples with an RNA integrity number (RIN) > 7.0 were used for subsequent processing. The average RIN for these samples was 8.3. Total RNA was subjected to two rounds of poly(A) selection using oligo d(T)_25_ magnetic beads (New England BioLabs, Ipswich, MA). Illumina-compatible RNA-seq libraries were prepared using a NEBNext Ultra RNA library prep kit for Illumina (New England BioLabs). cDNA libraries were purified using AMPure XP beads (Beckman Coulter, Brea, CA) and quantified using an Agilent Bioanalyzer and Qubit analysis. Equimolar amounts of barcoded libraries were pooled and sequenced on the NovaSeq6000 platform (Illumina, San Diego, CA) with single 101-bp reads in a 1 × 101 configuration. Samples had a minimum ~ 28 million read sequencing depth per sample.

### RNA-Seq Analysis

RNA-seq reads were imported into CLC Genomics Workbench (version 20.0.3, QIAGEN) for preliminary analysis using a modified version of the workflow for RNA-Seq analysis with export to IPA. All reads were batch-processed and mapped to the *Mus musculus* C57BL/6J (GRCm39) reference genome. Mapping settings for RNA-Seq Analysis tool were set to defaults including similarity fraction of 0.8. mRNA sequences between different strains within this species are similar enough (> 99%) such that mapping criteria are sufficient to match genes. Sequences obtained from each mouse strain and sample mapped at the same rate to the reference genome (mean percent matching ± standard deviation = 92.5 ± 2.5%). bTBI vs. sham samples were compared for each sex from all eight strains (e.g., male 129S1/SvImJ, female 129S1/SvImJ, etc.) using the Differential Analysis for RNA-Seq tool. Differentially expressed genes were considered significant if they met the following criteria: mean reads per kilobase of transcript per million mapped reads (RPKM) > 10.0, fold change in either direction ≥ 1.5, and p < 0.05.

### Pathway Analysis

Functional analyses were generated with the aid of Ingenuity Pathway Analysis (IPA) (QIAGEN). Core analysis was performed on the dataset generated by CLC based on RPKM values for genes that met criteria for significance, which generated lists of significant canonical pathways, associated diseases and functions, and differentially expressed genes. Comparison analysis was used to compare differential expression and affected pathways due to injury across strains within each sex. Canonical pathways were based on significant differentially expressed genes, and a pathway itself was considered significant if *p* < 0.05.

### VisuaL Annotation Display (VLAD) for Functional Gene Ontology

Functional themes based on GO annotations were identified and visualized using the VisuaL Annotation Display (VLAD) tool from the Mouse Genome Informatics (MGI) database (version 1.9.0, The Jackson Laboratory) [50–52]. VLAD allows for the simultaneous comparison of multiple query sets (query set = list of differentially expressed genes in bTBI vs. sham for one group) while reducing interior GO terms to distill information and preserve GO hierarchy. Analysis was limited to genes with functional annotations in the MGI database. Both male and female groups were input with both up- and downregulated genes included in each query set to simplify the visual output. The top 25 scoring terms across groups are included in Figure 9A-C. GO categories were considered significant if fold change in either direction was ≥ 1.5 and *p* < 0.05. VLAD output data including values for statistical significance and differentially expressed genes by group for each category are given in Online Resource 1.

### Statistics

Significant gene expression changes between groups were determined by the CLC Genomics Workbench Differential Expression tool, which performs a multi-factorial calculation with a negative binomial generalized linear model that adjusts for batch effects. Gene set enrichment analysis to identify affected gene ontology categories was conducted using the CLC Genomics Workbench Gene Set Test tool [53] based on ANOVA statistics that were summed for each category and adjusted for the number of genes in the category. Core pathway analysis in IPA involved a Fisher’s Exact Test considering the matches between the reference set and the observed genes that were not excluded by cutoffs. Pathways that were determined to have more genes than expected by chance were considered significant. Data for the UpSet plot, volcano plots, and heat maps were manipulated using the dplyr (version 1.1.4), reshape2 (version 1.4.4), data.table (version 1.15.4), and stringr (version 1.5.1) packages [54–57]. The UpSet plot was generated using the ComplexUpset package (version 1.3.3) [58–60]. The bar graph and heat maps were generated using the ggplot2 (version 3.5.1) package, and the volcano plots were created using the ggplot2 and ggrepel (version 0.9.5) packages [61, 62].

## Results

### Differential Gene Expression Patterns in the Mouse Hippocampus Due to bTBI Vary by Sex and Strain

**Fig. 1:**
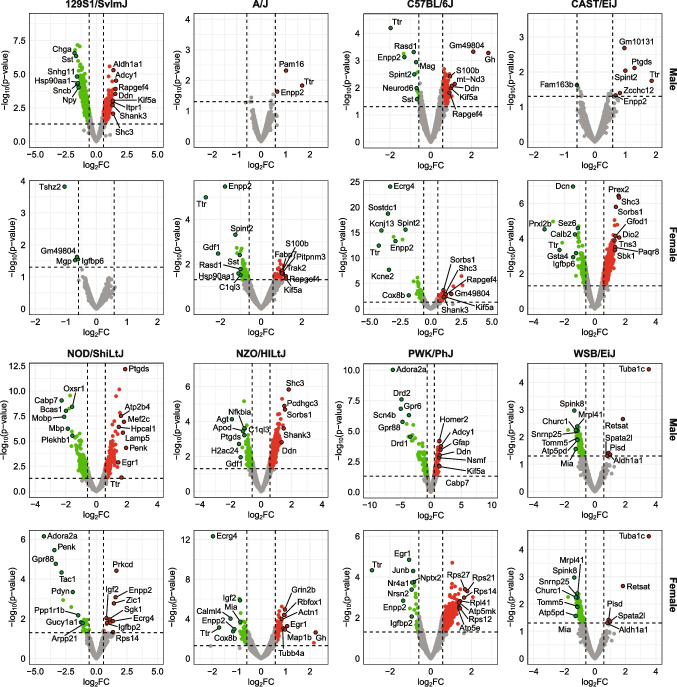
Top dysregulated genes due to bTBI by sex and strain. Volcano plots depict genes with a maximum group mean RPKM ≥ 10. Dotted lines indicate criteria for significance (*p* < 0.05, absolute value of fold change (FC) ≥ 1.5 or log_2_FC ≥ 0.585). Green (upper left area) = downregulated, red (upper right area) = upregulated, gray (central and lower areas) = no significant change

**Fig. 2:**
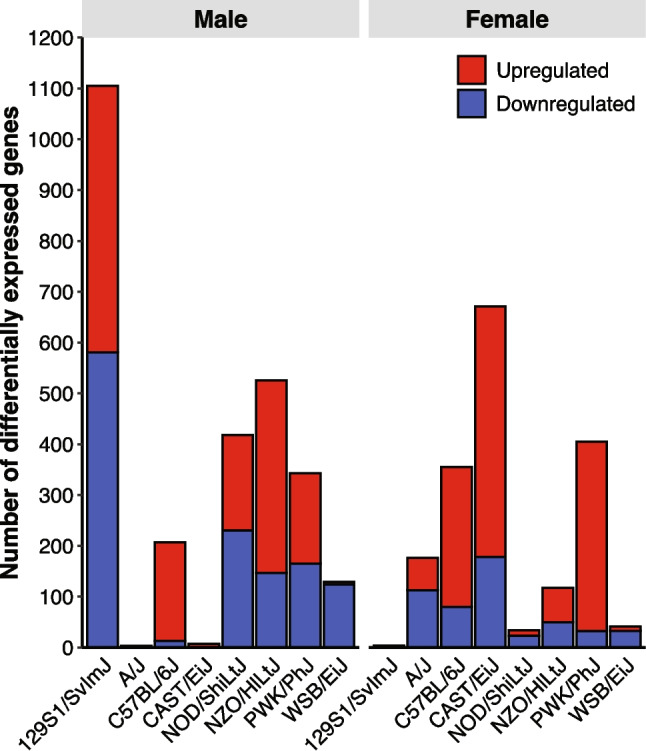
Direction of gene dysregulation due to bTBI by strain and sex. The column graph depicts the total number of upregulated (red, top) or downregulated (blue, bottom) genes due to bTBI in each group

**Fig. 3:**
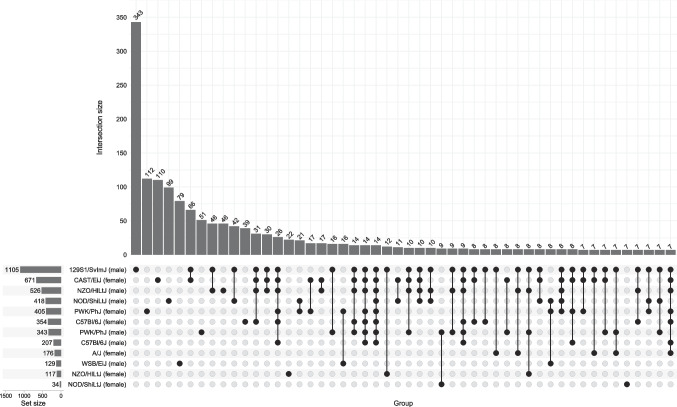
Visualization of overlaps in differential gene expression due to bTBI across strains and sexes. The UpSet plot displays commonalities in differential gene expression due to bTBI across sex and strain identified in bulk hippocampal RNA-seq analysis. Vertical columns indicate the exclusive intersection size, or the number of dysregulated genes that belong to the groups defining the intersection but not to any other set. Interaction size is displayed above each column. The dot matrix indicates intersections referenced in the column, the number of genes uniquely dysregulated in each sample group (•) or in common between different groups (•–•). Set size bars show the total number of dysregulated genes in bTBI vs. sham for each group. The top 50 interaction sets for relevant groups are shown

Figure 1 illustrates genes with upregulated and downregulated expression of hippocampal mRNA that meet stringent criteria of ≥ 1.5-fold change. The figure includes several example genes that are dysregulated across different groups, with all meeting the minimum fold change threshold and *p*-values ≤ 0.05. Approximately 11,000–13,000 genes were detected overall for each group, meaning a given gene showed a maximum group mean RPKM (i.e., greater mean in sham or bTBI condition) ≥ 1. We identified patterns in differential expression of individual genes in male and female mice from all eight strains and determined that the number of differentially expressed genes (DEGs) varied considerably by group under these criteria (Fig. 2). Male 129S1/SvImJ mice showed the highest degree of dysregulation with 1105 DEGs overall, 353 of which were unique to this group. In contrast, male WSB mice had relatively few DEGs (*n* = 129), but 59% were specific to this group (*n* = 76). The highest degree of similarity in differential expression profiles was between male 129S1/SvImJ and female CAST mice, which were also the two groups with the greatest degree of dysregulation overall. For 129S1/SvImJ mice, which showed the greatest sex-dependent differences within a strain, 11,424 genes were detected in females (*n* = 4 DEGs) and 13,413 genes were detected in males (*n* = 1105 DEGs), indicating that the large difference in DEGs did not correlate with the number of detected genes. Figure 3 depicts overlaps between different groups. There were no genes universally dysregulated across all groups, although four genes were dysregulated in 10 groups (*Enpp2*, *Ttr*, *Aldh1a1*, *Ppp1r3c*).

### bTBI Induces Hippocampal Dysregulation of Genes Related to Mitochondrial Function and Cellular Metabolism in Select Mouse Strains

**Fig. 4:**
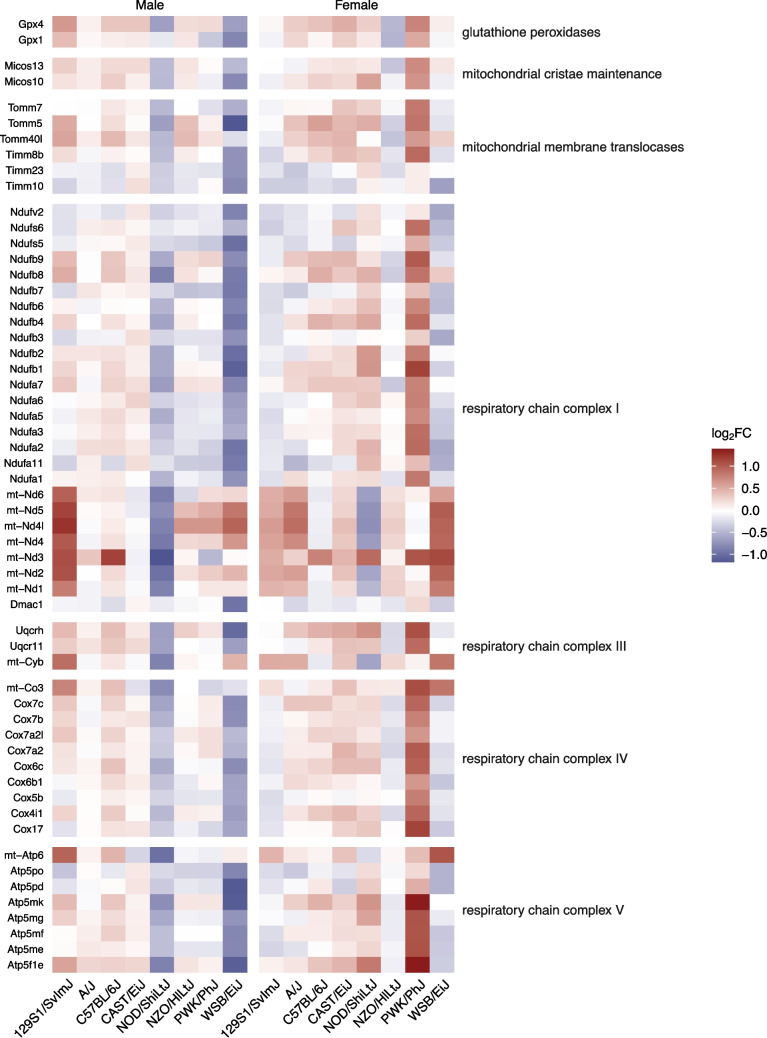
Differential expression of mitochondria-associated genes due to bTBI across sex and strain. The heat map shows log_2_ fold change of mRNA levels in bTBI vs. sham in male and female mice of eight strains. Red = upregulated, blue = downregulated

**Fig. 5:**
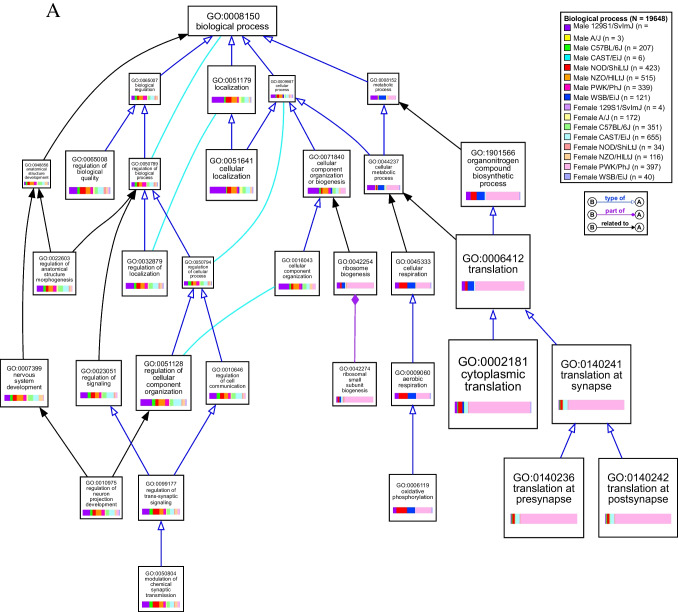

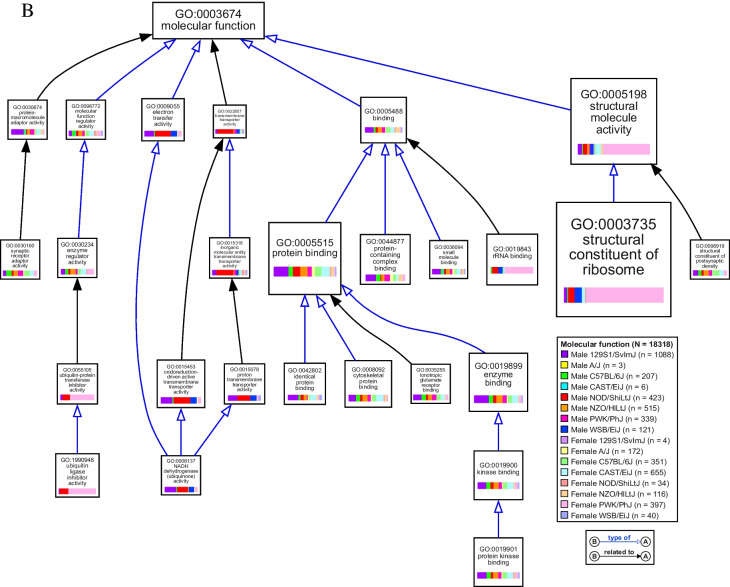

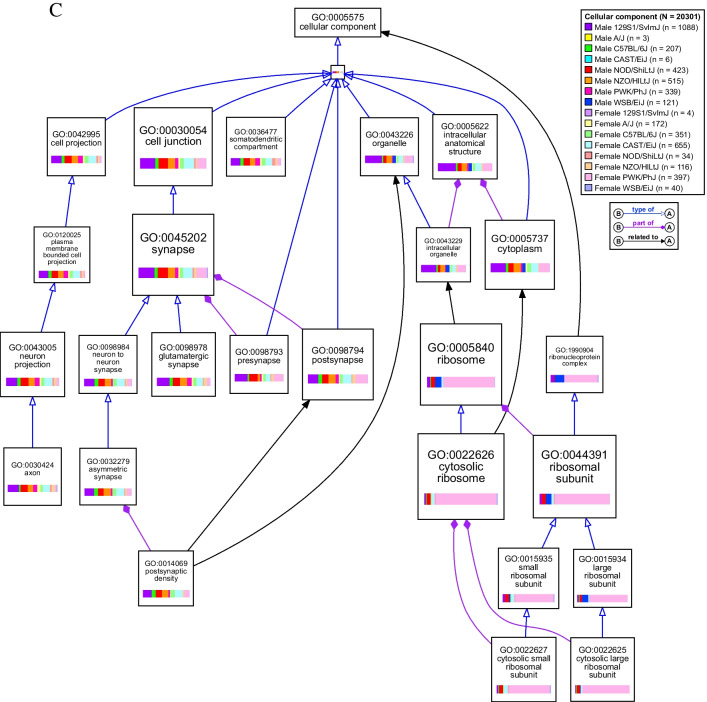
VisuaL Annotation Display (VLAD) of gene ontology (GO) term enrichment due to bTBI across sexes and strains. Top 25 scoring GO terms were mapped using the VLAD tool from MGI. Size of node represents score (larger node indicates greater statistical significance) with the minimum score from all groups used to draw the node. GO term identifiers and description of term are listed on each node. Colored bars in each node show the relative size of the significance score for each group. Lines connecting nodes indicate the relationship between two nodes: purple line with solid diamond = “part of”; blue line with hollow arrow = “is a”; black arrow = indirect relationship with interior nodes hidden. *N* = total number of genes and *n* = number of differentially expressed genes per group analyzed by VLAD. Diagrams show gene ontology terms related to (**a**) biological processes, (**b**) molecular functions, and (**c**) cellular components

**Fig. 6:**
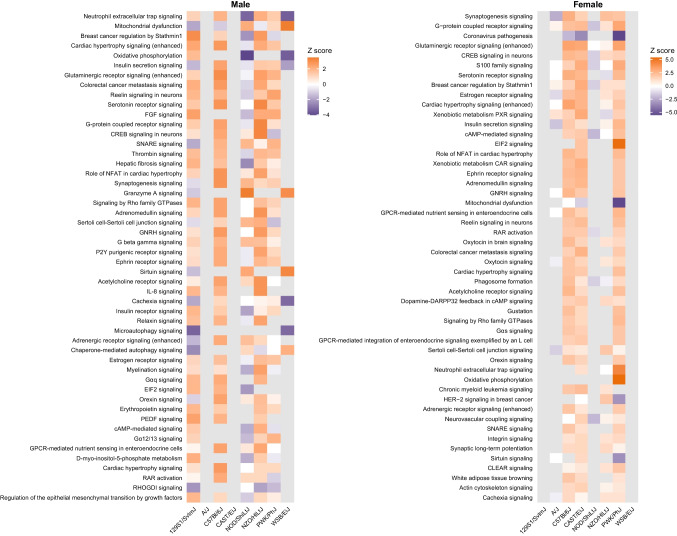
Affected canonical pathways due to bTBI in male and female mice of eight strains. Heat map depicts select canonical pathways identified using Ingenuity Pathway Analysis that were activated or repressed in sham vs. blast male and female mice across strains. Orange = activated, purple = repressed

In male WSB/EiJ mice, components of mitochondrial respiratory chain complex I (*Dmac1, Ndufa11*, *Ndufa2*, *Ndufb1*, *Ndufb2*, *Ndufb3*, *Ndufb4*, *Ndufb6*, *Ndufb7*, *Ndufs5*, *Ndufv2)*, complex III (*Uqcrh*, *Uqcrhl*), and complex V (*Atp5f1e*, *Atp5pd*, *Atp5po*) as well as mitochondrial membrane translocases (*Timm10*, *Timm23*, *Tomm5*) and glutathione peroxidase 1 (*Gpx1*) contributed to significant dysregulation of mitochondrial-related gene ontology (GO) terms and pathways (Fig. 4). Based on the percentage of dysregulated genes per category, male WSB/EiJ mice showed significant enrichment of GO terms confined to biological processes related to mitochondrial respiration, including proton motive force-driven mitochondrial ATP synthesis (GO:0042776, k = 12, *p* < 0.001), oxidative phosphorylation (GO:0006119, k = 13, *p* < 0.001), and mitochondrial respiratory chain complex I assembly (GO:0032981, k = 11, *p* < 0.001) (Fig. 5a). Similarly, male WSB/EiJ mice were limited to respiration-related molecular function categories, mainly stemming from the enrichment of genes involved in NADH dehydrogenase (ubiquinone) activity (GO:0008137, k = 3, *p* < 0.001) and cellular component terms related to mitochondrial structure, including mitochondrial inner membrane (GO:0005743, k = 37, *p* < 0.001) and mitochondrial protein-containing complex (GO:0098798, k = 30, *p* < 0.001) (Fig. 5b-c). Pathway analysis revealed suppression of oxidative phosphorylation (Z = −3.464, *p* < 0.001) and activation of mitochondrial dysfunction (Z = 3.464, *p* < 0.001) and sirtuin signaling (Z = 3.162, *p* < 0.001) pathways in male WSB/EiJ mice (Fig. 6).

Male NOD/ShiLtJ mice showed similar dysregulation of mitochondria-associated pathways (Fig. 6). However, these effects mainly involved downregulation of mitochondrial-encoded genes in complex I (*mt-Nd1*, *mt-Nd2*, *mt-Nd3*, *mt-Nd4*, *mt-Nd4l*, *mt-Nd5*, *mt-Nd6*), complex III (*mt-Cyb*), and complex V (*mt-Atp6*) and nuclear genes in complex III (*Uqcr11*, *Uqcrhl*) and complex V (*Atp5f1e*), as well as downregulation of nuclear genes in complex I (*Ndufa7*, *Ndufb4*, *Ndufb8*) and glutathione peroxidase 4 (*Gpx4*). In contrast, male 129S1/SvImJ showed dysregulation of the same pathways in opposite directions, including activation of oxidative phosphorylation (Z = 1.807, *p* < 0.001) and suppression of mitochondrial dysfunction (Z = −1.808, *p* < 0.001).

PWK/PhJ was the only strain among female mice that showed significant enrichment of GO terms and canonical pathways related to mitochondrial function. Dysregulated genes were related to mitochondrial respiratory complex I (*Ndufa1*, *Ndufa2*, *Ndufa3*, *Ndufa5*, *Ndufa6*, *Ndufa7*, *Ndufb1*, *Ndufb2*, *Ndufb4*, *Ndufb6*, *Ndufb8*, *Ndufb9*, *Ndufs6*, *mt-Nd3*), complex III (*Uqcr10*, *Uqcr11*, *Uqcrhl*, *Uqcrq*), complex IV (*Cox17*, *Cox4i1*, *Cox5b*, *Cox6b1*, *Cox6c*, *Cox7a2*, *Cox7a2l*, *Cox7b*, *Cox7c, mt-Co3*), and complex V (*Atp5f1e*, *Atp5me*, *Atp5mf*, *Atp5mg*, *Atp5mk*), mitochondrial cristae maintenance genes (*Micos10*, *Micos13*), and mitochondrial membrane translocases (*Timm8b*, *Tomm40l*, *Tomm5*, *Tomm7*) (Fig. 4). Female PWK/PhJ mice were also enriched in mitochondria-related GO terms, including proton motive force-driven ATP synthesis (GO:0042776, k = 18, *p* < 0.001), mitochondrial respirasome (GO:0005746, k = 28, *p* < 0.001), inner mitochondrial membrane protein complex (GO:0098800, k = 35, *p* < 0.001), respiratory chain complex (GO:0098803, k = 28, *p* < 0.001), as well as the unique term aerobic electron transport chain (GO:0019646, k = 16, *p* < 0.001) (Fig. 5a-c). Female PWK/PhJ mice showed a pathway pattern opposite to male WSB/EiJ mice: suppression of mitochondrial dysfunction (Z = −5.396, *p* < 0.001), sirtuin signaling (Z = −3.153, p < 0.001), and activation of oxidative phosphorylation (Z = 5.385, *p* < 0.001).

Dysregulation of mitochondrial genes also contributed to the activation or suppression of immune response pathways. For example, neutrophil extracellular trap (NET) signaling was activated in female PWK/PhJ mice (Z = 4.359, *p* < 0.001) and suppressed in male NOD/ShiLtJ (Z = −3.300, p < 0.001) and WSB/EiJ (Z = −3.464, *p* < 0.001) mice. Conversely, granzyme A signaling was activated in male NOD/ShiLtJ (Z = 3.464, *p* < 0.001) and WSB/EiJ (Z = 3.000, p < 0.001) mice but suppressed in female PWK/PhJ mice (Z = −4.000, *p* < 0.001) (Fig. 6).

### bTBI Induces Hippocampal Dysregulation of Genes Related to Translation and Ribosomal Structure in Select Groups

**Fig. 7:**
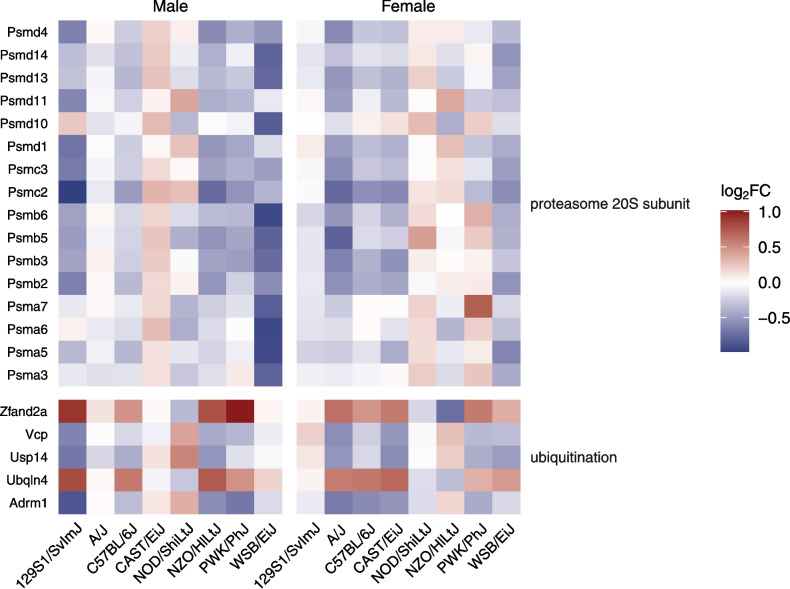
Differential expression of ribosome-associated genes due to bTBI across sex and strain. The heat map shows log_2_ fold change of mRNA levels in bTBI vs. sham in male and female mice of eight strains. Red = upregulated, blue = downregulated

Female PWK/PhJ mice were particularly enriched in GO categories related to translation, particularly due to dysregulation of many genes that comprised the small ribosomal subunit (*Fau*, *Rps3*, *Rps5*, *Rps7*, *Rps8*, *Rps9*, *Rps10*, *Rps11*, *Rps12*, *Rps13*, *Rps14*, *Rps15*, *Rps15a*, *Rps16*, *Rps17*, *Rps18*, *Rps19*, *Rps20*, *Rps21*, *Rps23*, *Rps24*, *Rps25*, *Rps26*, *Rps27*, *Rps27a*, *Rps28*, *Rps29*, *Rpsa*), large ribosomal subunit (*Rpl5*, *Rpl9*, *Rpl10a*, *Rpl11*, *Rpl12*, *Rpl13a*, *Rpl17*, *Rpl18a*, *Rpl19*, *Rpl22*, *Rpl23*, *Rpl23a*, *Rpl24*, *Rpl26*, *Rpl27*, *Rpl27a*, *Rpl29*, *Rpl30*, *Rpl31*, *Rpl32*, *Rpl34*, *Rpl35*, *Rpl35a*, *Rpl36*, *Rpl36a*, *Rpl36al*, *Rpl37*, *Rpl37a*, *Rpl38*, *Rpl39*, *Rpl41*, *Rplp1*, *Rplp2*, *Rps12*, *Uba52*, *Uba52rt*), and mitochondrial ribosome (*Chchd1*, *Mrpl23*, *Mrpl33*, *Mrpl53*, *Mrps18c*, *Ndufa7*) (Fig. 7). Ribosomal small subunit biogenesis (GO:0042274, k = 19, *p* < 0.001), large ribosomal subunit rRNA binding (GO:0070780, k = 4, *p* < 0.001), and the cytosolic small ribosomal subunit (GO:0022627, k = 28, *p* < 0.001) were affected only in female PWK/PhJ mice (Fig. 5a-c). Additional significant GO terms included translation at presynapse (GO:0140236, k = 37, *p* < 0.001), translation at postsynapse (GO:0140242, k = 37, *p* < 0.001), and cytoplasmic translation (GO:0002181, k = 58, *p* < 0.001), structural constituent of ribosome (GO:0003735, k = 69, *p* < 0.001), and cytosolic large ribosomal subunit (GO:0022625, k = 36, *p* < 0.001) (Fig. 5a-c).

A smaller set of ribosomal genes were also differentially expressed in female CAST/EiJ mice (*Mrpl33*, *Mt3*, *Rnf10*, *Rpl12*, *Rpl26*, *Rpl35a*, *Rpl35rt*, *Rpl39*, *Rpl9*, *Rps12*, *Rps14*, *Rps18*, *Rps21*, *Rps23*, *Rps24*, *Rps27*, *Rps29*) (Fig. 7). Translation at presynapse (k = 8, *p* < 0.001), translation at postsynapse (k = 8, *p* < 0.001), cytoplasmic translation (k = 13, *p* < 0.001), structural constituent of ribosome (k = 14, *p* = 0.001), and cytosolic large ribosomal subunit (k = 7, p = 0.0096) were also affected in female CAST/EiJ mice (Fig. 5a-c). Translation at presynapse (k = 5, *p* = 0.002), translation at postsynapse (k = 5, p = 0.002), and cytosolic large ribosomal subunit were significant in male NOD/ShiLtJ mice due to dysregulation of *Rpl22*, *Rpl35a*, *Rpl5*, *Rps14*, and *Rps27*. Genes related to the mitochondrial ribosome were also dysregulated in male 129S1/SvImJ (*Mrpl2*, *Mrpl21*, *Mrpl33*, *Mrpl36*, *Mrpl38*, *Mrpl4*, *Mrps11*, *Mrps12*, *Mrps18b*, *Mrps34*) and male WSB/EiJ (*Chchd1*, *Mrpl11*, *Mrpl13*, *Mrpl14*, *Mrpl28*, *Mrpl41*, *Mrpl46*, *Mrpl53*, *Mrps18c*, *Mrps33*) mice (Fig. 7). rRNA binding was significantly affected in male NOD/ShiLtJ (k = 7, *p* < 0.001) and WSB/EiJ (k = 3, *p* = 0.007) and female PWK/PhJ (k = 17, *p* < 0.001) mice (Fig. 5b).

EIF2 signaling was a top-scoring pathway across both male and female strain comparisons and was significantly activated in male 129S1/SvImJ (Z = 1.941, *p* < 0.001) and C57BL/6J (Z = 2.000, *p* = 0.01) and female CAST/EiJ (Z = 2.121, *p* < 0.001) and PWK/PhJ (Z = 5.292, *p* < 0.001) mice. In contrast, EIF2 signaling was suppressed in male NOD/ShiLtJ mice (Z = −2.121, *p* < 0.001). Regulation of eIF4 and p70S6K signaling was also activated in male 129S1/SvImJ mice (Z = 1.633, *p* = 0.01) (Fig. 6).

### bTBI Induces Hippocampal Dysregulation of Genes Related to Protein Degradation and Ubiquitination in Several Mouse Strains

**Fig. 8:**
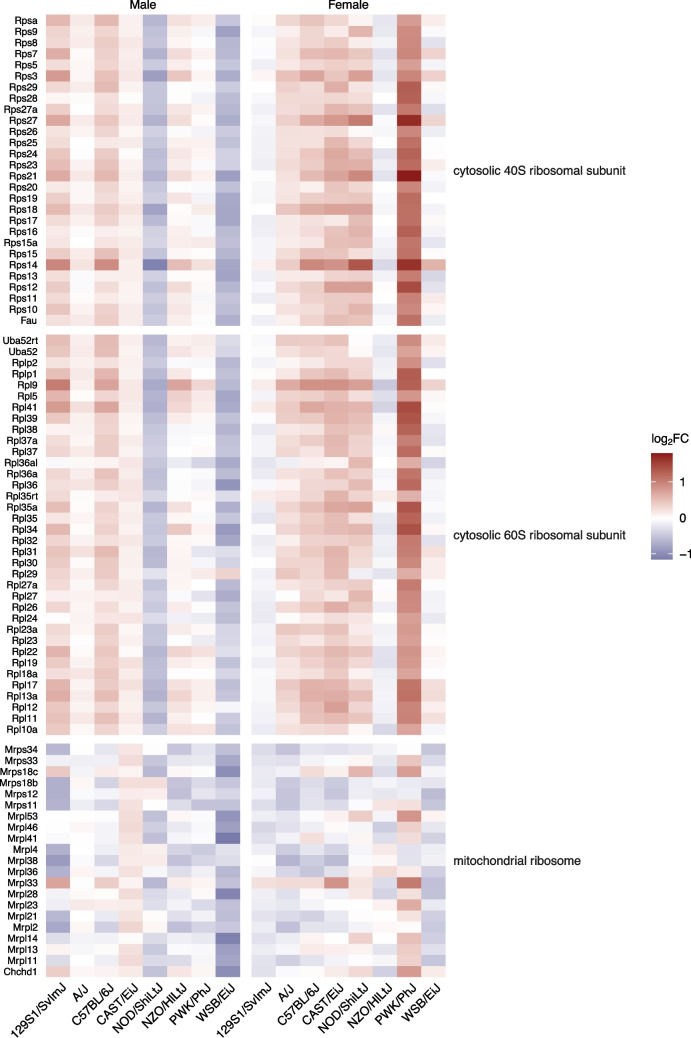
Differential expression of genes associated with protein degradation due to bTBI across sex and strain. Heat map shows log_2_ fold change of mRNA levels in bTBI vs. sham in male and female mice of eight strains. Red = upregulated, blue = downregulated

Pathways related to protein degradation were affected in male WSB/EiJ mice due to dysregulation of proteasome 20S subunit genes (*Psma3*, *Psma5*, *Psma6*, *Psma7*, *Psmb3*, *Psmb5*, *Psmb6*, *Psmd10*, *Psmd13*, *Psmd14*) including chaperone-mediated autophagy signaling (Z = 2.121, *p* = 0.0165), cachexia signaling (Z = −3.162, *p* < 0.001), and microautophagy signaling (Z = −3.162, *p* < 0.001) (Figs. 6, 8). Other proteasome-associated genes were dysregulated in male 129S1/SvImJ mice (*Adrm1*, *Psmb2*, *Psmc2*, *Psmc3*, *Psmc4*, *Psmd1*, *Psmd11*, *Psmd4*, *Psmd6*, *Ubqln4*, *Usp14*, *Vcp*, *Zfand2a*), resulting in suppression of cachexia signaling (Z = −1.768, *p* < 0.001) (Figs. 6, 8). GO terms for proteasome complex (GO:0000502) and proteasome-mediated ubiquitin-dependent protein catabolic process (GO:0043161) were significantly affected in male 129S1/SvImJ and WSB/EiJ mice (Fig. 5c). Ubiquitin ligase inhibitor activity (GO:1,990,948) was enriched in male NOD/ShiLtJ (k = 3, *p* < 0.001) and female PWK/PhJ (k = 7, *p* < 0.001) mice (Fig. 5b).

### bTBI Induces Hippocampal Dysregulation of Genes Related to Synaptic Integrity and Signaling in Several Mouse Strains

**Fig. 9:**
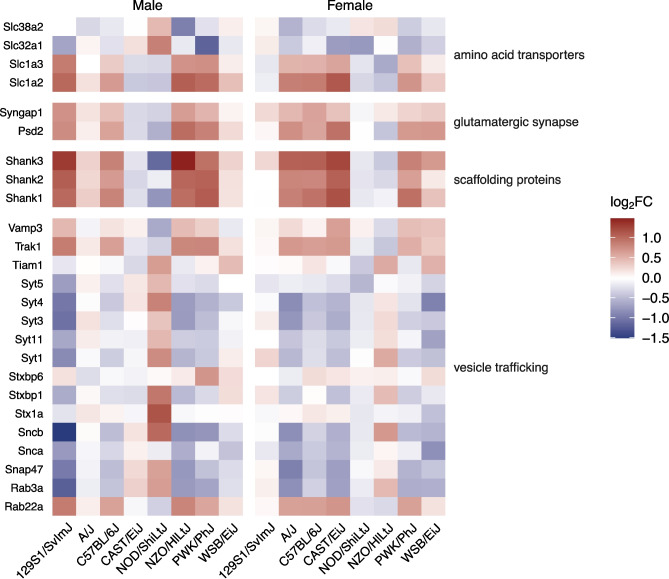
Differential expression of synaptic genes due to bTBI across sex and strain. Heat map shows log_2_ fold change of mRNA levels in bTBI vs. sham in male and female mice of eight strains. Red = upregulated, blue = downregulated

Genes related to synaptic plasticity, structure, and signaling (e.g., encoding amino acid transporters, scaffolding proteins, proteins involved in vesicle trafficking) were dysregulated widely across mouse strains in the hippocampus following bTBI (Fig. 9). Modulation of chemical synaptic transmission (GO:0050804) was significantly enriched in 10 out of 16 groups due to bTBI, including male 129S1/SvImJ (k = 100, *p* < 0.001), C57BL/6J (k = 26, *p* < 0.001), NOD/ShiLtJ (k = 52, *p* < 0.001), NZO/HlLtJ (k = 49, *p* < 0.001), PWK/PhJ (k = 32, *p* < 0.001) mice and female A/J (k = 14, *p* = 0.003), C57BL/6J (k = 35, *p* < 0.001), CAST/EiJ (k = 57, *p* < 0.001), NOD/ShiLtJ (k = 5, *p* = 0.008), and PWK/PhJ (k = 34, *p* < 0.001) mice (Fig. 5a). The GO term for glutamatergic synapse (GO:0098978) was enriched in 11 groups, including male 129S1/SvImJ (k = 117, *p* < 0.001), C57BL/6J (k = 25, *p* < 0.001), NOD/ShiLtJ (k = 58, *p* < 0.001), NZO/HlLtJ (k = 60, *p* < 0.001), and PWK/PhJ (k = 40, *p* < 0.001) and female A/J (k = 19, *p* < 0.001), C57BL/6J (k = 42, *p* < 0.001), CAST/EiJ (k = 82, *p* < 0.001), NOD/ShiLtJ (k = 6, *p* = 0.002), NZO/HlLtJ (k = 24, *p* < 0.001), and PWK/PhJ (k = 44, *p* < 0.001) (Fig. 5c). Genes linked to the glutamatergic synapse that were differentially expressed in six or more groups included *Actn1*, *Adcy1*, *Agap2*, *Apba1*, *Apc*, *Arhgap33*, *Camk2a*, *Crtc1*, *Dlgap3*, *Fam107a*, *Git1*, *Grk3*, *Homer2*, *Map2*, *Myo6*, *Neurl1a*, *Nsmf*, *Psd2*, *Psd3*, *Rapgef4*, *Rims1*, *Rims4*, *Shank1*, *Shank2*, *Shank3*, *Sipa1l1*, *Slc1a2*, *Tspoap1*, *Ube3b*, *Vgf*, and *Wasf3* (Fig. 9). Ionotropic glutamate receptor binding (GO:0035255) was also dysregulated in male 129S1/SvImJ (k = 15, *p* < 0.001), C57BL/6J (k = 5, *p* < 0.001), NOD/ShiLtJ (k = 4, *p* = 0.02), NZO/HlLtJ (k = 6, *p* = 0.003), and PWK/PhJ (k = 6, *p* < 0.001) (Fig. 5b). Additionally, the glutaminergic receptor signaling pathway was activated in male C57BL/6J (Z = 3.000, *p* = 0.002) and NZO/HlLtJ mice (Z = 2.558, *p* < 0.001) and female C57BL/6J (Z = 3.317, *p* = 0.008), CAST/EiJ (Z = 3.128, *p* < 0.001), and PWK/PhJ (Z = 3.317, *p* = 0.02) mice (Fig. 6).

Canonical pathways related to other neurotransmitters that were also activated due to bTBI included acetylcholine receptor signaling in male (Z = 2.449, *p* = 0.006) and female (Z = 2.646, *p* = 0.02) C57BL/6J mice and serotonin receptor signaling in male C57BL/6J (Z = 2.840, *p* < 0.001), male NZO/HlLtJ (Z = 3.128, *p* < 0.001), female C57BL/6J (Z = 2.840, *p* = 0.003), female CAST/EiJ (Z = 2.646, *p* < 0.001), and female PWK/PhJ mice (Z = 2.496, *p* = 0.04). The orexin signaling pathway was also activated in male C57BL/6J (Z = 2.449, *p* = 0.02), male NZO/HlLtJ (Z = 1.732, *p* = 0.005), female C57BL/6J (Z = 2.121, *p* = 0.02), and female CAST/EiJ (Z = 1.500, *p* < 0.001) mice. The endocannabinoid neuronal synapse pathway was suppressed only in male PWK/PhJ mice (Z = −1.633, *p* = 0.001) (Fig. 6).

As a cellular component, genes related to the dendrite (GO:0030425) were significantly enriched in male 129S1/SvImJ (k = 117, *p* < 0.001), C57BL/6J (k = 33, *p* < 0.001), NOD/ShiLtJ (k = 55, *p* < 0.001), NZO/HlLtJ (k = 64, *p* < 0.001), and PWK/PhJ (k = 46, *p* < 0.001) mice and female A/J (k = 15, *p* = 0.002), C57BL/6J (k = 43, *p* < 0.001), CAST/EiJ (k = 75, *p* < 0.001), NOD/ShiLtJ (k = 9, *p* < 0.001), NZO/HlLtJ (k = 17, *p* < 0.001), and PWK/PhJ (k = 45, *p* < 0.001) mice. Six genes associated with this term were dysregulated across 8 groups (*Camk2a*, *Ddn*, *Nsmf*, *Ppp1r9b*, *Shank1*, *Trak2*) (Fig. 9). Several terms linked to the structure and function of the synapse were also enriched, including structural constituent of postsynaptic density (GO:0098919) in male 129S1/SvImJ (k = 6, *p* < 0.001), C57BL/6J (k = 3, *p* < 0.001), NOD/ShiLtJ (k = 2, *p* = 0.02), NZO/HlLtJ (k = 5, *p* < 0.001), and PWK/PhJ (k = 3, *p* < 0.001) mice and female C57BL/6J (k = 6, *p* < 0.001), CAST/EiJ (k = 5, *p* < 0.001), and PWK/PhJ (k = 3, *p* < 0.001) mice. Postsynaptic density (GO:0014069) was also enriched in male 129S1/SvImJ (k = 76, *p* < 0.001), C57BL/6J (k = 23, *p* < 0.001), NOD/ShiLtJ (k = 37, *p* < 0.001), NZO/HlLtJ (k = 43, *p* < 0.001), and PWK/PhJ (k = 20, *p* < 0.001) and female C57BL/6J (k = 32, *p* < 0.001), CAST/EiJ (k = 56, *p* < 0.001), NZO/HlLtJ (k = 11, *p* < 0.001), PWK/PhJ (k = 38, *p* < 0.001) mice, as well as presynapse (GO:0098793) in male 129S1/SvImJ (k = 110, *p* < 0.001), C57BL/6J (k = 17, *p* < 0.001), NOD/ShiLtJ (k = 58, *p* < 0.001), NZO/HlLtJ (k = 38, *p* < 0.001), and PWK/PhJ (k = 26, *p* < 0.001) and female A/J (k = 17, *p* < 0.001), C57BL/6J (k = 32, *p* < 0.001), CAST/EiJ (k = 67, *p* < 0.001), NOD/ShiLtJ (k = 6, *p* = 0.002), NZO/HlLtJ (k = 18, *p* < 0.001), and PWK/PhJ (k = 67, *p* < 0.001) mice. Synaptic receptor adaptor activity (GO:0030160) was affected in male 129S1/SvImJ (k = 4, *p* < 0.001), C57BL/6J (k = 4, *p* < 0.001), NOD/ShiLtJ (k = 2, *p* = 0.002), NZO/HlLtJ (k = 4, *p* < 0.001), PWK/PhJ (k = 4, *p* < 0.001) and female A/J (k = 1, *p* = 0.03), C57BL/6J (k = 4, *p* < 0.001), CAST/EiJ (k = 4, *p* < 0.001), and PWK/PhJ (k = 3, *p* < 0.001) mice (Fig. 5a-c).

Synaptogenesis signaling was activated in male C57BL/6J (Z = 2.828, p < 0.001) and NOD/ShiLtJ (Z = 1.886, *p* < 0.001) and female CAST/EiJ (Z = 1.606, *p* < 0.001), NZO/HlLtJ (Z = 2.236, *p* = 0.004), and PWK/PhJ (Z = 2.121, *p* = 0.03) mice but suppressed in female A/J mice (Z = −2.236, p = 0.03). SNARE signaling was activated in male C57BL/6J (Z = 2.000, *p* = 0.03) and NOD/ShiLtJ (Z = 1.941, *p* < 0.001) and female PWK/PhJ (Z = 1.633, *p* = 0.02) mice but suppressed in male 129S1/SvImJ (Z = −1.698, *p* < 0.001) mice (Fig. 6). Significant dysregulation of genes related to the regulation of neuron projection development (GO:0010975) was observed in male 129S1/SvImJ (k = 80, *p* < 0.001), C57BL/6J (k = 20, *p* < 0.001), NOD/ShiLtJ (k = 32, *p* < 0.001), NZO/HlLtJ (k = 46, *p* < 0.001), and PWK/PhJ (k = 27, p < 0.001) mice and in female C57BL/6J (k = 28, *p* < 0.001), CAST/EiJ (k = 54, *p* < 0.001), NZO/HlLtJ (k = 12, *p* < 0.001), and PWK/PhJ (k = 28, *p* < 0.001) mice (Fig. 5a). Reelin signaling in neurons was activated in male 129S1/SvImJ (Z = 1.941, *p* = 0.004), male C57BL/6J (Z = 2.000, *p* = 0.03), male PWK/PhJ (Z = 2.449, *p* = 0.01), and female CAST/EiJ (Z = 2.333, *p* = 0.001) mice (Fig. 6).

### bTBI Induces Hippocampal Dysregulation of Genes Related to G Protein Signaling in Several Mouse Strains

**Fig. 10:**
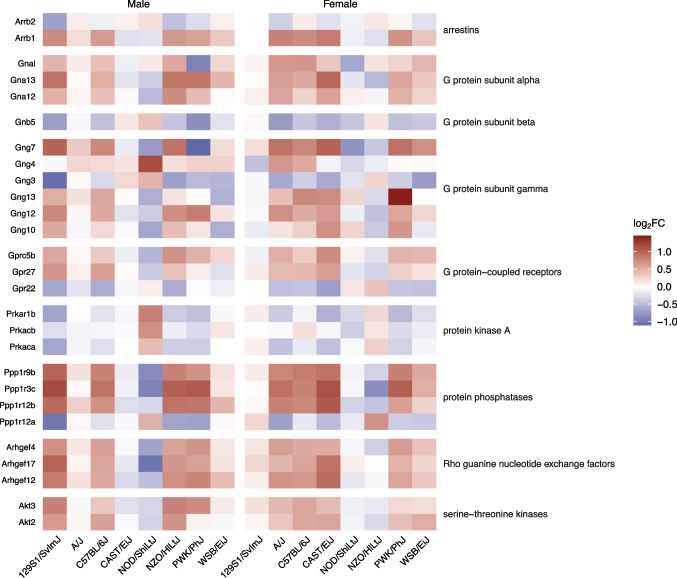
Differential expression of genes associated with intracellular signaling due to bTBI across sex and strain. Heat map shows log_2_ fold change of mRNA levels in bTBI vs. sham in male and female mice of eight strains. Red = upregulated, blue = downregulated

Figure 10 displays dysregulated genes linked to G protein signaling. G-protein coupled receptor (GPCR) signaling was activated in male C57BL/6J (Z = 2.500, *p* < 0.001), male NZO/HlLtJ (Z = 3.157, *p* < 0.001), female C57BL/6J (Z = 2.236, *p* = 0.003), and female CAST/EiJ mice (Z = 2.401, *p* = 0.001) and suppressed in female NOD/ShiLtJ mice (Z = −1.633, *p* < 0.001). cAMP-mediated G protein signaling pathways were dysregulated in some groups following bTBI. G_αs_ signaling was activated in male (Z = 2.000, *p* = 0.02) and female (Z = 2.000, *p* = 0.01) C57BL/6J mice and in female CAST/EiJ mice (Z = 1.633, *p* < 0.001). G_αi_ signaling was activated only in male 129S1/SvImJ mice (Z = 1.667, *p* < 0.001). The cAMP-mediated signaling pathway was suppressed in male (Z = −1.667, *p* = 0.02) and female (Z = −2.000, *p* < 0.001) NOD/ShiLtJ mice. Phosphatidylinositol-mediated G protein signaling was also somewhat dysregulated, particularly G_αq_ signaling in male 129S1/SvImJ (Z = 1.897, *p* = 0.01), C57BL/6J (Z = 2.236, *p* = 0.01), and NZO/ShiLtJ (Z = 2.000, *p* = 0.01) mice. Signaling by Rho family GTPases was activated in male 129S1/SvImJ (Z = 2.000, *p* = 0.003), male C57BL/6J (Z = 2.449, p = 0.008), male NZO/HlLtJ (Z = 1.667, p = 0.002), and female C57BL/6J (Z = 1.890, *p* = 0.005) mice, while RhoGDI signaling was suppressed in male 129S1/SvImJ (Z = −2.138, *p* = 0.003) and NZO/HlLtJ (Z = −1.890, *p* = 0.003) mice. Other G protein pathways that were significantly affected included activation of G_α12/13_ signaling in male PWK/PhJ (Z = 2.000, *p* = 0.01) and female CAST/EiJ (Z = 1.633, *p* = 0.03) mice, as well as activation of G_βγ_ signaling in male C57BL/6J (Z = 2.000, *p* < 0.001), NOD/ShiLtJ (Z = 1.732, p < 0.001), and NZO/HlLtJ (Z = 1.667, *p* < 0.001) mice (Fig. 6).

## Discussion

### Sex and Strain Affect Hippocampal Gene Expression Changes After bTBI

In this study, we identified variations in hippocampal transcriptomic profiles of male and female mice from eight genetically distinct strains (129S1/SvImJ, A/J, C57BL/6J, CAST/EiJ, NOD/ShiLtJ, NZO/HlLtJ, PWK/PhJ, WSB/EiJ) at 1 month following primary blast exposure. Our dataset can also be used to identify how dysregulations in individual genes previously implicated in TBI can vary by sex and genotype. For example, we found that the gene encoding the Aβ scavenger transthyretin (*Ttr*) was dysregulated by blast exposure in the majority (10) of the injured groups. *Ttr* was significantly upregulated across multiple hippocampal cell types following fluid percussion injury (FPI) and in microglia following repetitive CHI in C57BL/6J mice [63, 64]. Conversely, we observed that *Ttr* was downregulated due to bTBI in both male and female C57BL/6J mice, although it was upregulated in several other strains. Ectonucleotide pyrophosphatase/phosphodiesterase 2 (*Enpp2*) was identified as a key contributor to changes in the mitochondrial respirasome and was upregulated in microglia following rCHI in C57BL/6J mice [64]. *Enpp2* is a target in Alzheimer’s disease (AD) known to induce GPCR signaling and mediate inflammation [65]. *Enpp2* was dysregulated in 10 groups in our dataset but again was downregulated in C57BL/6J due to bTBI rather than upregulated. Glial fibrillary acidic protein (*Gfap*) upregulation is a well-established sign of reactive astrocytosis in rodent models following bTBI and other types of neurotrauma [66]. We found that *Gfap* expression was upregulated in 6 groups after injury, including male 129S1/SvImJ, male C57BL/6J, male NZO/HlLtJ, male PWK/PhJ, female CAST/EiJ, and female PWK/PhJ mice. Interestingly, *Gfap* expression was significantly downregulated in male NOD/ShiLtJ mice. Decreased GFAP protein expression in the hippocampus and amygdala has been reported previously in a Sprague Dawley (SD) rat model of repetitive low-level bTBI as a sign of widespread loss of gliovascular connections [67, 68]. We hypothesize that the observed variability in the direction of gene dysregulation as well as the number of DEGs across groups suggests differences in transcriptional regulation in response to bTBI which may be sex- and/or genotype-dependent and that these differences may provide preliminary insight into susceptibility or resistance to injury.

Some dysregulated genes identified by our analysis were directly associated with TBI comorbidities and symptoms. For example, neuropeptide Y (*Npy*) was differentially expressed in 7 groups following bTBI, with the majority being downregulated. Reduced hippocampal NPY expression has been reported previously in rodent models of TBI: in a rat model of severe CCI, NPY^+^ cells in CA3 were chronically decreased in the contralateral hippocampus compared to shams [69]. Similarly, NPY expression was reduced in the CA1, CA3, and dentate gyrus (DG) subregions of the hippocampus following low-level blast exposure using an exploding wire model in SD rats [70]. Clinically, low levels of NPY in plasma and cerebrospinal fluid (CSF) have been suggested as a pathophysiological component of post-traumatic stress disorder (PTSD) [71–73]. The relationship between PTSD and bTBI pathology and their overlapping symptoms have been reported in the literature since the advent of military blast exposure over a century ago [74–77]. In the rodent hippocampus, decreased levels of NPY may be associated with impaired cognition, and *Npy* knockout mice have demonstrated anxiety-like behavior in the open field test (OFT), elevated plus maze (EPM), and light–dark box [71]. A separate study using the exploding wire model reported a PTSD-like phenotype in blast-exposed rats, including impaired performance on EPM and increased acoustic startle response (ASR) magnitude [78]. In a shock tube model of repetitive mild bTBI, injured rats displayed anxiety-like behavior in the elevated zero maze (EZM), potentiated ASR, and enhanced freezing in cued fear conditioning [79]. It is possible that bTBI could induce downregulation of NPY in the CNS in some mouse strains, possibly contributing to anxiety- or PTSD-like symptoms, and warrants further investigation. More broadly, our RNA-seq data can be integrated with existing literature on TBI-associated behaviors as well as ongoing phenotypic analyses across mouse strains.

### Mitochondrial Respiratory Chain Complexes and Other Mitochondrial Signaling

Focusing on broader functional themes through gene ontology (GO) and pathway analyses, we concentrated on the top themes, pathways, and genes across strains and have identified several that may be relevant for further investigation. We found that a significant number of genes and pathways related to mitochondrial function and oxidative phosphorylation were differentially expressed in male 129S1/SvImJ, male NOD/ShiLtJ, male WSB/EiJ, and female PWK/PhJ mice. Complexes I (NADH dehydrogenase), III (cytochrome c reductase), and V (ATP synthase) were downregulated in male WSB/EiJ mice, complexes I, III, IV (cytochrome c oxidase), and V were downregulated in male NOD/ShiLtJ mice, and complexes I, III, IV, and V were upregulated in female PWK/PhJ mice. Male 129S1/SvImJ exhibit mixed effects with both up and downregulation of complex I and V genes and upregulation of complexes III and IV. Interestingly, male WSB/EiJ mice were particularly susceptible to mitochondrial dysfunction but not dysregulation of other genes, whereas the other three groups showed enrichment of other terms and pathways in addition to those related to metabolism.

Oxidative stress and mitochondrial dysfunction are key components of acute and subacute secondary TBI pathophysiology [80, 81]. Markers of acute mitochondrial dysfunction, such as decreased levels of ATP and mitochondrial glutamate oxaloacetate transaminase (GOT2) in human neuroblastoma (SH-SY5Y) cells and cortical tissue from C57BL/6J mice, have been reported at 6 h post-bTBI at lower BOPs [82]. However, impaired mitochondrial function and glucose hypometabolism can persist for days or weeks after TBI [83–85]. Following CHI, C57BL/6J mice had decreased hippocampal complex I-driven electron transport and ATP synthesis capacity at 1-month post-injury, suggesting dysregulation of at least complexes I and V is likely present in other TBI models [86]. Dysregulation of complex I-V was also reported at 7 days post-FPI in the hippocampus and/or cortex of SD rats [87]. Additional studies have focused on the specific effects of bTBI on mitochondrial function. In active-duty service members exposed to moderate blasts during a 10-day military training program, several mitochondrial-associated genes were dysregulated in blood samples collected 3 days post-exposure [88]. Interestingly, mitochondrial respiration was impaired in synaptic but not glial-enriched mitochondria isolated from the prefrontal (PFC) and amygdala/entorhinal/piriform (AEP) cortex of SD rats at 3 days post-bTBI [89]. In C57BL/6J mice, low-intensity open field blast exposure caused decreased complex III and V activity and increased complex I activity at 30 days post-injury, as well as dysregulation of other mitochondria-associated proteins including GPX4, which was dysregulated in male 129S1/SvImJ, male NOD/ShiLtJ, and female PWK/PhJ mice in our data set [90]. Ultrastructural damage to mitochondria in several regions including the hippocampus was also observed in this model and persisted at 30 days post-injury [91]. Although these strains have not been compared in TBI, differences in mitochondrial gene regulation have been observed in the hypothalamus in male WSB/EiJ compared to C57BL/6J mice in response to a high-fat diet [92]. Thus, our observations of blast-induced mitochondrial dysfunction in the hippocampus are supported by findings in the literature and seem sex- and strain-dependent.

### Neutrophil Extracellular Trap Signaling

Neutrophil extracellular trap (NET) signaling showed the greatest cumulative Z-score in our male comparison analysis, indicating that this pathway was significantly dysregulated across multiple groups. NETs are composed of extracellular DNA, histones, and antimicrobial proteins which are released from activated neutrophils to target pathogens as well as other types of immune cells [93, 94]. We found that NET signaling was activated in female PWK/PhJ mice and suppressed in male NOD/ShiLtJ and WSB/EiJ mice in the hippocampus at 30 days post-bTBI. NETs have recently been identified as molecular drivers of neuroinflammation and cell death following TBI, ischemia/stroke, spinal cord injury (SCI), and AD [93, 95–99]. NET formation after TBI was associated with tissue hypoxia and cerebrovascular dysfunction in a mouse CCI model, and circulating NETs were found in patients with severe TBI [100]. In a rat model of diffuse axonal injury, NETs within the paraventricular nucleus (PVN) promoted formation of M1 (pro-inflammatory) microglia after TBI [101]. NET inhibition improved short-term neurological function and reduced cerebrovascular dysfunction in CCI mice and ameliorated neuroinflammation and neuronal apoptosis [102]. Thus, the role of NETs in the neuroimmune response to bTBI and potential genetic drivers of NET-associated pathology warrants additional study.

### Protein Processing

We found that genes encoding ribosomal proteins were dysregulated in male NOD/ShiLtJ, female CAST/EiJ, and female PWK/PhJ mice, and components of the mitochondrial ribosome were dysregulated in male 129S1/SvImJ and WSB/EiJ mice in the mouse hippocampus following bTBI. Additionally, rRNA binding was significantly affected in male NOD/ShiLtJ, male WSB/EiJ, and female PWK/PhJ mice due to injury. Degradation of 28S rRNA has been reported in the ipsilateral cortex and hippocampus in rats following FPI [103]. Ribosomal dysfunction has also been implicated in early AD pathology: decreased protein synthesis and downregulation of 5.8S and 5S rRNA were reported in the inferior parietal lobule and superior middle temporal gyri of mild cognitive impairment (MCI) and AD brains [104]. Although TBI-induced transcriptional changes in ribosomal proteins have not been reported previously in rodent models, we found that these differences were only present in strains that are not typically used in preclinical TBI models. Interestingly, several ribosomal genes including *RPL6*, *RPL35*, *MRPL50*, *MRPL1*, *MRPL3*, and *MRPL46* were dysregulated in blood samples from active-duty military service members 3 days after moderate blast exposure [88].

Dysfunction of the ubiquitin proteasome system has been implicated in the pathology of several neurodegenerative diseases associated with reactive oxygen species (ROS) or aggregation of proteotoxic proteins such as AD, Parkinson’s disease (PD), amyotrophic lateral sclerosis (ALS), and Huntington’s disease (HD) [105, 106]. We observed downregulation of components of the 20S proteasome in male WSB/EiJ and 129S1/SvImJ mice due to bTBI, with additional genes related to ubiquitination dysregulated in male 129S1/SvImJ mice. Additionally, genes related to ubiquitin ligase inhibitor activity were differentially expressed in male NOD/ShiLtJ and female PWK/PhJ mice after injury. Proteasome activation has been suggested as a possible therapeutic mechanism for the treatment of proteotoxic diseases including AD, PD, and TBI through J2 prostaglandins, NRF2 activation, and other mechanisms [107–110]. The proteolytically active 20S proteasome core can also function via a ubiquitin-independent pathway which breaks down intrinsically disordered proteins such as amyloid beta (Aβ), tau, TAR DNA-binding protein 43 (TDP-43), and α-synuclein, which are known to accumulate in various neurodegenerative disorders. Decreased proteasome activity, driven in part by downregulation of the 20S proteasome, has been postulated to contribute to neurodegeneration by reducing the clearance of pathogenic proteins; consequently, the activation of 20S proteasome has been suggested as a therapeutic target for neurodegenerative diseases [111]. Recently, dihydroquinazolines were shown to enhance 20S proteasome activity and induce degradation of α-synuclein in vitro, supporting the role of the 20S proteasome as a therapeutic target for neurodegenerative diseases [112]. Based on our data, we hypothesize that the therapeutic effects of proteasome activation in TBI mouse models may vary by genotype.

### Synaptic Signaling and Integrity

Damage to neuronal synapses and dendritic spines is suggested to play a role in bTBI pathology due to stretching and shearing [113]. We observed wide dysregulation of genes associated with synaptic signaling, vesicle trafficking, and pre- and postsynaptic integrity in male and/or female mice from most strains due to bTBI. For example, SH3 and multiple ankyrin repeat domains 1, 2, and 3 (*Shank1*, *Shank2*, *Shank3*), which encode scaffold proteins in the glutamatergic postsynaptic density, were upregulated in 7 groups, including male and female C57BL/6J mice. In a CCI model using male C57BL/6J mice, *Shank3* expression began to increase in microglia around 30 days and was significantly upregulated by 90 days post-injury, whereas *Shank3* knockout prevented microglial proliferation in the hippocampus after CHI in mice [114, 115]. We also observed dysregulation of several synaptotagmins (*Syt1*, *Syt3*, *Syt4*, *Syt5*, *Syt11*), which are thought to mediate calcium binding to trigger neurotransmitter release at the synapse, in up to 5 groups due to bTBI. SYT1 protein levels were increased by open field blast at 24 h post-injury in C57BL/6J mice; however, we only observed upregulation of *Syt1* in male NOD/ShiLtJ mice at 1-month post-injury, and *Syt1* was downregulated in male 129S1/SvImJ mice [116]. Overexpression of synaptotagmin in the substantia nigra is associated with PD and has been reported on the ipsilateral side after CCI in SD rats [117]. Increased proteolysis of synaptotagmin has also been observed in the ipsilateral cortex of SD rats at 48 h post-CCI [118]. Interestingly, synaptotagmin family genes (*SYT1*, *SYT4*, *SYT5*, *SYT7*, and *SYT13*) were downregulated in the anterior temporal lobe in chronic traumatic encephalopathy (CTE), CTE/AD, and AD post-mortem human brains [119].

Blast injury has been shown to reduce hippocampal long-term potentiation (LTP) via disruption of synaptic proteins [120]. In male Long Evans rats, dendritic degeneration and decreased levels of synaptic proteins were observed in CA1 at 13 months post-blast [121]. Increased total numbers of excitatory synapses have been reported in the hippocampus of male C57BL/6J mice at 30 days post-blast and in CA1 in a rat model of CCI at 60 days post-injury [122, 123]. Microstructural changes in postsynaptic density and a decrease in active zone length were also observed in male C57BL/6J mice at 30 days post-injury [122]. We have also previously reported that open field blast injury decreases dendritic length in the hippocampus of C57BL/6J mice [124]. Notably, while synaptic genes in C57BL/6J, NOD/ShiLtJ, and PWK/PhJ mice of both sexes were highlighted in our data set by VLAD, we found that several strains showed sex differences in these GO categories, including 129S1/SvImJ (male only), A/J (female only), CAST/EiJ (female only), and NZO/HlLtJ (male only). Together, these results are consistent with bTBI-induced disruption of hippocampal synaptic integrity and signaling in most mouse strains at 1-month post-injury but suggest that these effects may vary in a sex- and/or strain-dependent manner.

### Rho GTPases

Rho GTPases function as regulators of morphological neuroplasticity, particularly dendritic spine morphogenesis and axonal outgrowth [125]. Dysregulation of Rho family GTPases has been implicated in neurodegenerative diseases including AD, PD, HD, ALS, spinal cord injury, and ischemia/reperfusion injury [126]. We found that signaling by Rho family GTPases was activated in several groups after bTBI, including male 129S1/SvImJ, male C57BL/6J, male NZO/HlLtJ, and female C57BL/6J (Z = 1.890, p = 0.005) mice, while RhoGDI signaling was suppressed only in male 129S1/SvImJ and NZO/HlLtJ mice. Upregulation of RhoA and B was observed in human brains proximal to lesion after TBI beginning at 12–48 h post-injury and persisting for several months [127]. Similarly, RhoA was activated by 3 days post-FPI in both ipsilateral and contralateral hippocampus and cortex in SD rats [128]. Pharmacological RhoA-ROCK inhibition was shown to improve cognitive and motor deficits and prevent TBI-induced dendritic spine remodeling in CA1 pyramidal neurons following CCI in male C57BL/6 mice [129]. Dysregulation of signaling by Rho GTPases further emphasizes that genetic background may affect hippocampal synaptic plasticity following bTBI.

### Genetic Effects on Response to TBI

A significant challenge in identifying specific processes involved in the brain’s response to injury is the considerable variability in symptoms and pathology within injured populations. Recent emphasis regarding the influence of genetic factors on neurotrauma has focused on single nucleotide polymorphisms (SNPs) that correlate with differential outcomes after TBI. For example, the Arg72Pro (rs1042522) SNP of *TP53*, which encodes the cell cycle regulator p53, is associated with improved initiation of apoptosis in vitro and with poorer outcomes up to 24 months post-TBI in homozygous patients [130–132]. Apolipoprotein E (*APOE*) has garnered significant interest regarding its role in dementia; numerous studies have also linked the *APOE* ε4 allele with increased mortality, increased damage, slower recovery rate, and poorer long-term outcomes following brain injury in both humans and animal models of TBI [133]. In rodents, *APOE* ε4 has been shown to impair blood–brain barrier repair and contribute to the development of tau hyperphosphorylation following injury [25, 134]. Variable recovery outcomes following brain injury have also been shown to result from polymorphisms in other genes, including those coding for cytokines (e.g., *TNF*, *IL1RN*, *IL6*, *TGFB1*), DNA repair (e.g., *PARP1*), B-cell lymphoma 2 (*BCL2*), brain-derived neurotropic factor (*BDNF*), nociceptin opioid peptide (NOP) receptor (*OPRL1*), and serotonin receptor 1A (*HTR1A*) [18, 135–141].

While significant progress has been made in understanding risks and outcomes for TBI from an epidemiological perspective, there is still considerable work to be done to incorporate biologically relevant genetic variation in clinical research and preclinical animal models [142]. Race and ethnicity are often omitted as variables in clinical studies on TBI, and women and racial/ethnic minorities are still underrepresented in many clinical trials on TBI [143, 144]. Little to no research has been conducted into the effects of genetic and biological variations on TBI pathophysiology in the context of race and ethnicity, although these factors are known to influence oxidative stress and inflammation [145]. However, one study reported that a history of TBI accelerated AD onset in a race/ethnicity and gender-dependent manner, suggesting that these factors may be critical for advancing our understanding of post-TBI sequelae and potential chronic neurodegenerative effects [146]. Most of these studies have investigated mechanical TBIs in the civilian population, and there has been little study of genetic influences on bTBI in military personnel. Furthermore, relatively few animal studies have examined strain differences in TBI models rather than individual mutations, making it difficult to consider the interactions between genotypic variations that would be present in patients [28, 30, 31, 147]. Interestingly, a recent study using male mice of 12 strains from the recombinant inbred Collaborative Cross panel reported significant strain-dependent differences in spatial learning and memory in Barnes maze at 13–20 days post-lateral FPI, suggesting that genotype can play a role in hippocampal-associated behavioral outcomes after TBI [148]. Recent advancements in neurotrauma research have increasingly focused on sex as a biological variable in preclinical models, yet the interaction between sex and genotype remains largely unexplored [27, 39, 40, 64, 149–151]. Here, we found that genotype and sex did influence the degree and type of differential gene expression following blast injury, suggesting that further analysis of the effect of these variables on functional outcome and neuropathology are warranted. Thus, our study substantiates our hypothesis that sex and genotype may impact transcriptomic and molecular changes after neurotrauma with the aim of increasing translational potential for preclinical blast and TBI models.

## Conclusions

We found that differential gene expression in the mouse hippocampus varied at 1-month post-injury in a strain and sex-dependent manner. This dataset represents a foundational knowledge to consider sex and genotype interactions in bTBI. Future directions include phenotypic and behavioral comparisons within the context of genetic analysis to identify putative mechanistic and therapeutic factors.

## Supplementary Information

Below is the link to the electronic supplementary material.

Supplementary file1 (XLSX 15677 KB)

## Data Availability

The datasets generated and analyzed during the current study are available through the NCBI Gene Expression Omnibus (GEO) repository (GSE285890).

## Declarations

### Disclaimer

The contents do not represent the views of the United States Government, and the opinions, interpretations, conclusions and recommendations are those of the authors and are not necessarily endorsed by the Department of Veterans Affairs or the Department of Defense.
